# Effect of pharmacological selectivity of SGLT2 inhibitors on cardiovascular outcomes in patients with type 2 diabetes: a meta-analysis

**DOI:** 10.1038/s41598-024-52331-w

**Published:** 2024-01-25

**Authors:** Alex Ali Sayour, Attila Oláh, Mihály Ruppert, Bálint András Barta, Béla Merkely, Tamás Radovits

**Affiliations:** Heart and Vascular Center, Department of Cardiology, Semmelweis University, Városmajor Str. 68, 1122 Budapest, Hungary

**Keywords:** Cardiology, Endocrinology, Diabetes

## Abstract

Sodium–glucose cotransporter 2 (SGLT2) inhibitors reduce major adverse cardiovascular events (MACE) in type 2 diabetic (T2DM) patients. Pharmacological selectivity of these agents to SGLT2 over SGLT1 is highly variant, with unknown clinical relevance. Genetically reduced SGLT1—but not SGLT2—activity correlates with lower risk of heart failure and mortality, therefore additional non-selective SGLT1 inhibition might be beneficial. In this prespecified meta-analysis, we included 6 randomized, placebo-controlled cardiovascular outcome trials of SGLT2 inhibitors assessing MACE in 57,553 patients with T2DM. Mixed-effects meta-regression revealed that pharmacological selectivity of SGLT2 inhibitors (either as continuous or dichotomized variable) had no significant impact on most outcomes. However, lower SGLT2 selectivity correlated with significantly lower risk of stroke (pseudo-R^2^ = 78%; p = 0.011). Indeed, dual SGLT1/2 inhibitors significantly reduced the risk of stroke (hazard ratio [HR], 0.78; 95% confidence interval [CI], 0.64–0.94), unlike selective agents (p for interaction = 0.018). The risk of diabetic ketoacidosis and genital infections was higher in both pharmacological groups versus placebo. However, hypotension occurred more often with non-selective SGLT2 inhibitors (odds ratio [OR], 1.87; 95% CI, 1.20–2.92) compared with selective agents (p for interaction = 0.044). In conclusion, dual SGLT1/2 inhibition reduces stroke in high-risk T2DM patients but has limited additional effect on other clinical outcomes.

## Introduction

Sodium–glucose cotransporter 2 (SGLT2) inhibitors were originally designed to aid glucose control in patients with diabetes mellitus by blocking SGLT2 in the proximal convoluted tubule of the kidneys, resulting in glucosuria. However, their antidiabetic effect turned out to be modest^1^, rather, these medications consistently reduced the relative risk of hospitalization for heart failure (HF) in these type 2 diabetic patients across all cardiovascular outcome trials, but some clinical endpoints were heterogeneous^2–7^. Later it became clear that SGLT2 inhibitors exert salutary cardiovascular effects independently of the presence of diabetes, nonetheless, the mechanism of action is currently incompletely understood^8–10^.

Of note, SGLT2 inhibitors show substantial variance in pharmacological selectivity to SGLT2 over SGLT1^1,11,12^. Whereas sotagliflozin is considered to be a dual SGLT1/2 inhibitor, canagliflozin also shows clinically relevant renal, gastrointestinal, and myocardial SGLT1 inhibitory effect at therapeutic plasma concentrations^13–19^. On the other hand, the SGLT2 inhibitors empagliflozin, dapagliflozin, and ertugliflozin are highly selective to SGLT2. These pharmacodynamic differences might be clinically relevant given the fact that SGLT1 is responsible for the majority of glucose absorption from the gastrointestinal system and contributes to postprandial glucose excursions^20,21^, which phenomenon is well-known risk factor for adverse cardiovascular events^22^. Furthermore, humans with alterations in the gene encoding SGLT1 resulting in production of functionally limited transporter (resembling pharmacological SGLT1 inhibition) are protected from the development of HF, and all-cause death is significantly lower as compared with non-affected subjects^23^. On the contrary, those with functionally limited SGLT2 (resembling pharmacological SGLT2 inhibition) derive no meaningful benefit pertinent to these outcomes^24^. Additionally, preclinical studies have linked SGLT1 to the pathophysiology of HF^25–28^, as well as myocardial^29^, cerebral^30,31^, and renal ischemic injury^32^. Therefore, additional pharmacological inhibition of SGLT1 might have significant clinical implications on top of SGLT2 blockade in high-risk type 2 diabetic patients, which have not been established yet.

In this meta-analysis, our goal was to quantify the contribution of pharmacological SGLT2 selectivity to clinical efficacy and safety outcomes in type 2 diabetic patients treated with SGLT2 inhibitors, according to large-scale cardiovascular outcomes trials.

## Materials and methods

### Protocol and registration

The present meta-analysis was conducted and reported in compliance with the Preferred Reporting Items for Systematic Reviews and Meta-analyses (PRISMA) reporting guideline^33^. The protocol was prespecified and published on PROSPERO (registration no.: CRD42021273914).

### Eligibility criteria

*Population*: We selected randomized, placebo-controlled cardiovascular outcome trials assessing major adverse cardiovascular events (MACE: composite of cardiovascular death, nonfatal myocardial infarction [MI], and nonfatal stroke) with SGLT2 inhibitors in adult patients with type 2 diabetes mellitus. Trials enrolling patients without diabetes were excluded. We considered peer-reviewed, English-language publications without date restriction.

*Intervention*: The intervention constituted treatment with an SGLT2 inhibitor (empagliflozin, canagliflozin, dapagliflozin, ertugliflozin, or sotagliflozin).

*Comparator*: Placebo was considered as the comparator in all cases.

*Outcomes*: The main outcomes included MACE (composite of cardiovascular death, nonfatal MI, and nonfatal stroke); cardiovascular death; fatal and nonfatal stroke; fatal and nonfatal MI; hospitalization for HF; all-cause mortality; and renal composite endpoint. All trials reported total fatal and nonfatal stroke (regardless of subtype), except for the DECLARE-TIMI 58 trial, which reported ischemic stroke events only (included also in the composite of MACE in that trial)^4^. The primary and secondary outcomes, and definition of the renal composite endpoint are contained within Table 1.

**Table 1:** Participants and characteristics of included clinical trials.

	EMPA-REG OUTCOME 2015^2^	CANVAS Program 2017^3^	DECLARE-TIMI 58 2018^4^	CREDENCE 2019^5^	VERTIS CV 2020^6^	SCORED 2020^7^
Study design	Interventional, randomized, parallel-assigned, double blind, placebo-controlled (placebo vs. 10 mg or 25 mg empagliflozin)	Interventional, randomized, parallel-assigned, quadruple blind, placebo-controlled (placebo vs. 100 mg or 300 mg canagliflozin)	Interventional, randomized, parallel-assigned, double blind, placebo-controlled (placebo vs. 10 mg dapagliflozin)	Interventional, randomized, parallel-assigned, double blind, placebo-controlled (placebo vs. 100 mg canagliflozin)	Interventional, randomized, parallel-assigned, double blind, placebo-controlled (placebo vs. 5 mg or 15 mg ertugliflozin)	Interventional, randomized, parallel-assigned, quadruple blind, placebo-controlled (placebo vs. 200 mg or 400 mg sotagliflozin)
Pharmacological selectivity of tested medication (SGLT2:SGLT1), ratio	Empagliflozin: ~ 2700	Canagliflozin: ~ 260	Dapagliflozin: ~ 1200	Canagliflozin: ~ 260	Ertugliflozin: ~ 2200	Sotagliflozin: ~ 20
No. of participants	7020	10,142	17,160	4401	8246	10,584
Median follow-up, years	3.1	2.4	4.2	2.6	3.0	1.3
Age—mean ± SD, years	63 ± 9	63 ± 8	64 ± 7	63 ± 9	64 ± 8	69 ± 8
Male sex—no. (%)	5016 (71.5%)	6509 (64.2%)	10,738 (62.6%)	2907 (66.1%)	5769 (70.0%)	5830 (55%)
Race: White—no. (%)	5081 (72.4%)	7944 (78.3%)	13,653 (79.6%)	2931 (66.6%)	7240 (87.8%)	8749 (82.7%)
Baseline HbA1C—mean ± SD, %	8.1 ± 0.8	8.2 ± 0.9	8.3 ± 1.2	8.3 ± 1.3	8.2 ± 1.0	8.4 ± 1.3
Baseline eGFR—mean ± SD, mL/min/1.73 m^2^	74 ± 22	77 ± 21	85 ± 16	56 ± 18	76 ± 21	44 ± 11
Key inclusion criteria	Type 2 diabetes mellitus; > = 18 y–o; HbA1C = 7.0‒10.0% if on background therapy; HbA1C = 7.0‒9.0% if no background therapy; high CV risk	Type 2 diabetes mellitus; > = 30 y-o; HbA1C = 7.0‒10.5%; high CV risk	Type 2 diabetes mellitus; > = 40 y-o; HbA1C 6.5‒12.0%; high CV risk	Type 2 diabetes mellitus; > = 30 y-o; HbA1C = 6.5‒12.0%; eGFR = 30‒90 mL/min/1.73 m^2^ *AND* albuminuria; on ACEI or ARB	Type 2 diabetes mellitus; > = 40 y-o; HbA1C = 7.0‒10.5%; high CV risk	Type 2 diabetes mellitus; > = 18 y-o; HbA1C > = 7%; eGFR = 25‒60 mL/min/1.73 m^2^; high CV risk
Key exclusion criteria	eGFR < 30 mL/min/1.73 m^2^	eGFR < 30 mL/min/1.73 m^2^	CrCl < 60 mL/min	eGFR < 30 mL/min/1.73 m^2^	eGFR < 30 mL/min/1.73 m^2^	eGFR < 25 mL/min/1.73 m^2^
Primary outcomes	3-P MACE (excluding silent MI)	3-P MACE	Co-primary: 3-P MACE (with nonfatal ischemic stroke); CVD *AND* HHF	Renal composite #1 (ESRD *AND* doubling of serum creatinine level *AND* renal death *AND* CVD)	3P-MACE	CVD *AND* HHF *AND* urgent visits for HF (original co-primary: 3P-MACE; CVD *AND* HHF)
Key secondary outcomes	4-P MACE (3-P MACE *AND* hospitalization for UA); renal composite (doubling of serum creatinine level with eGFR < 45 mL/min/1.73 m^2^ *AND* dialysis *AND* renal death)	All-cause mortality; CVD; progression of albuminuria; CVD *AND* HHF; renal composite (> = 40% decrease in eGFR *AND* dialysis *AND* renal death)	Renal composite #1 (> = 40% decrease in eGFR *AND* ESRD *AND* renal death *AND* CVD); all-cause mortality; renal composite #2 (> = 40% decrease in eGFR *AND* ESRD *AND* renal death)	CVD *AND* HHF; 3-P MACE; HHF; renal composite #2 (ESRD *AND* doubling of serum creatinine level *AND* renal death); CVD; all-cause mortality; MACE *AND* HHF *AND* hospitalization for UA	CVD *AND* HHF; CVD; renal composite (renal death *AND* dialysis/transplant *AND* doubling of serum creatinine level)	Total no. of HHF *AND* urgent visits for HF; CVD; all-cause mortality; 3-P MACE *AND* HHF; CVD *AND* HHF *AND* urgent visits for HF *AND* no. of HF events during hospitalization; renal composite (50% decline in eGFR *AND* dialysis *AND* renal transplantation *AND* eGFR < 15 mL/min/1.73 m^2^); 3P-MACE
Link to trial registry	NCT01131676	NCT01032629; NCT01989754	NCT01730534	NCT02065791	NCT01986881	NCT03315143

When trials defined more than one renal composite endpoint, for the meta-analysis we used the one that was devoid of cardiovascular death (CVD) ensuring more homogeneity across studies.

*3P-MACE* three-point major adverse cardiovascular events comprising cardiovascular death, nonfatal myocardial infarction, and nonfatal stroke, *ACEI* angiotensin-converting enzyme inhibitor, *ARB* angiotensin receptor blocker, *CrCl* creatinine clearance, *CV* cardiovascular, *CVD* cardiovascular death, *eGFR* estimated glomerular filtration rate, *ESRD* end-stage renal disease, *HbA1C* glycated hemoglobin, *HHF* hospitalization for heart failure, *MI* myocardial infarction, *SD* standard deviation, *UA* unstable angina.

### Literature search, study selection, data collection, and quality assessment

The search terms are provided as Supplementary material. Two collaborators independently assessed the publications in line with the predefined selection criteria as outlined above. Disagreement was resolved by the senior author. For each involved trial, data were extracted on trial design, baseline characteristics of study populations, and outcomes. The Cochrane Risk of Bias Tool^34^ was used to evaluate individual study quality.

### Statistical analysis

The hazard ratios (HRs) and their 95% confidence intervals (CIs) were extracted for the binary prespecified outcomes and pooled using the Sidik–Jonkman^35^ random-effects model. Statistical heterogeneity (referred to as heterogeneity) was assessed using the Cochran Q homogeneity test, Higgins and Thompson I^2^, and Tau^2^. As per the Cochrane Handbook for Systematic Reviews of Interventions^36^, we considered the following for heterogeneity: I^2^ = 0% to 40% might not be important; I^2^ = 30–60% may represent moderate heterogeneity; I^2^ = 50–90% may represent substantial heterogeneity; I^2^ = 75–100% is considerable heterogeneity.

We performed mixed-effects meta-regression (based on the Sidik–Jonkman method) to assess the influence of pharmacological receptor selectivity (SGLT2:SGLT1 selectivity ratio as a continuous variable^1,11,12^) of each individual medication on the given outcome (yielding pseudo-R^2^ values, which describes the proportion of heterogeneity explained by this factor). Additionally, analyses were carried out by dichotomizing studies based on whether the studied medication has clinically relevant SGLT1 inhibitory effect (i.e. agents with low pharmacological SGLT2 selectivity: canagliflozin, sotagliflozin) or has no clinically meaningful SGLT1 inhibitory property (i.e. agents with high pharmacological SGLT2 selectivity: empagliflozin, dapagliflozin, ertugliflozin). In each case, mixed-effects meta-regression (based on the Sidik–Jonkman method) was used to assess difference between the pooled estimates of low vs. high SGLT2 selectivity groups, pertinent to each outcome.

Evaluation of reported adverse events (including all severe adverse events) was performed by extracting odd ratios (ORs) and their 95% CI (SGLT2 inhibitor treatment versus placebo) from the included trial. Then, the influence of pharmacological selectivity on these safety outcomes, either as dichotomous or continuous variable, was computed as described above.

All statistical analyses were carried out in Stata 17.0 (StataCorp LLC, College Station, TX, USA). A two-tailed p < 0.05 was considered statistically significant.

### Sensitivity analysis

Sensitivity analysis was carried out post hoc. Fatal and nonfatal stroke as an outcome was analyzed in individual study subgroups with baseline estimated glomerular filtration rate (eGFR) lower than 60 mL/min/1.73 m^2^ according to a previous meta-analysis^37^. The effect of selectivity of SGLT2 inhibitors on these subgroups was investigated as outlined above.

## Results

### Eligible studies, patient characteristics, and risk of bias

We identified an overall of 6 placebo-controlled randomized cardiovascular outcome trials (EMPA-REG OUTCOME^2^, CANVAS Program^3^, DECLARE-TIMI 58^4^, CREDENCE^5^, VERTIS CV^6^, and SCORED^7^) of three selective SGLT2 inhibitors (empagliflozin^2^, dapagliflozin^4^, and ertugliflozin^6^) and two SGLT2 inhibitors with clinically relevant SGLT1-inhibitory property (canagliflozin^3,5^ and sotagliflozin^7^), including a total of 57,553 patients with type 2 diabetes mellitus and high cardiovascular risk (Table 1). The mean (± standard deviation) age of all trial participants was 65 ± 8 years; 36,769 (63.9%) were men, and 20,784 (36.1%) were women; and 45,598 (79.2%) were White (Table 1). Across the 6 trials, the median follow-up ranged from 1.3 to 4.2 years, average HbA1C ranged from 8.1 to 8.3%, baseline average eGFR ranged from 44 to 85 mL/min/1.73 m^2^ (Table 1).

Supplementary materials contain the search terms, whereas Supplementary Fig. 1 depicts the selection process. All included trials showed low overall risk of bias according to the Cochrane Risk of Bias Tool^34^ (Supplementary Table 1); the SCORED trial^7^ ended prematurely due to loss of funding, therefore the primary endpoint was changed during the trial and investigator-reported events were used for endpoint analyses.

### Outcomes

#### Efficacy

**Figure 1:**
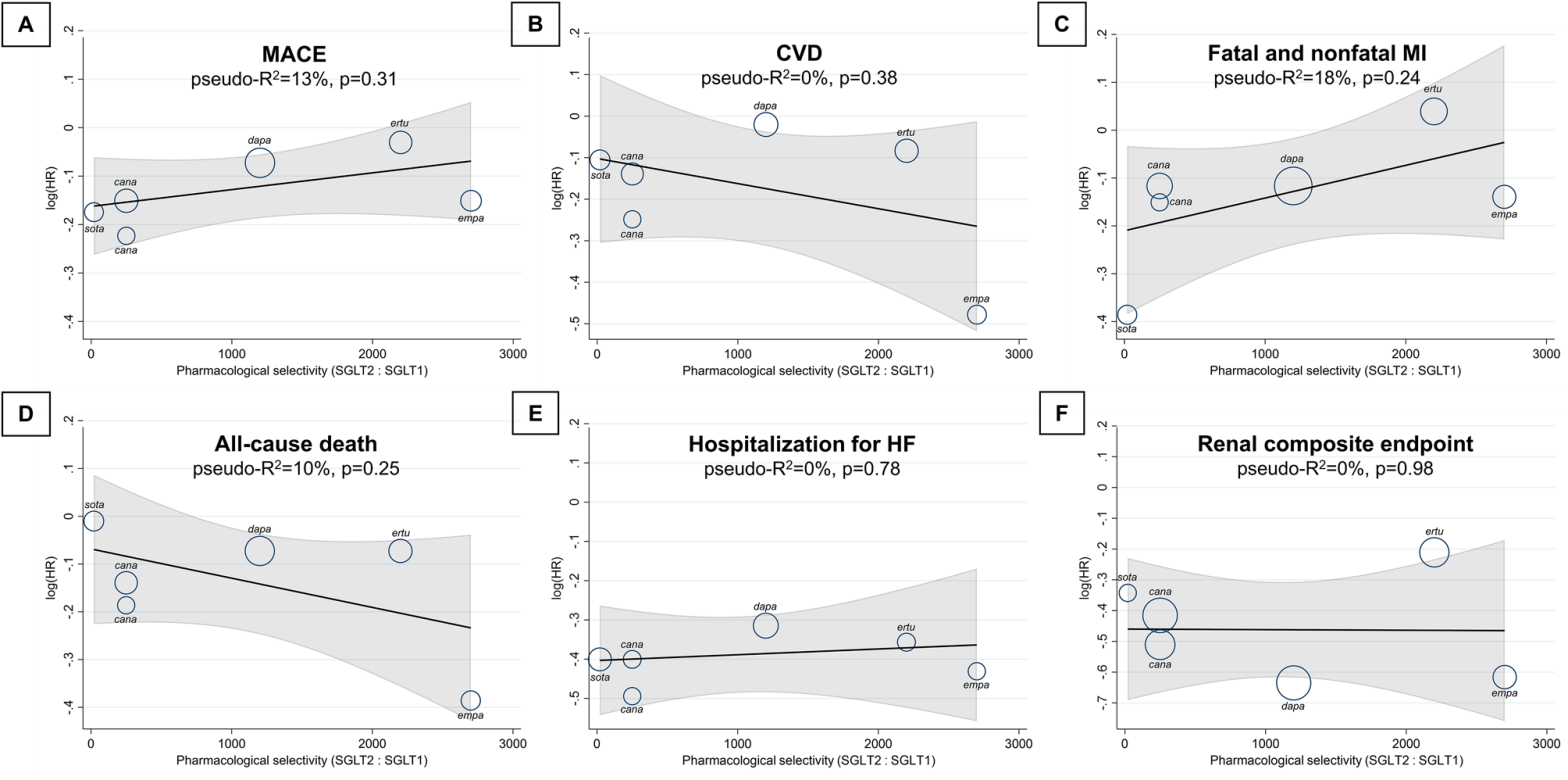
Effect of pharmacological selectivity of SGLT2 inhibitors on risk of MACE (**A**), CVD (**B**), fatal and nonfatal MI (**C**), all-cause death (**D**), hospitalization for HF (**E**), and renal composite endpoint (**F**) using the SGLT2:SGLT1 pharmacological selectivity ratio as continuous explanatory variable in a mixed-effects meta-regression analysis. *CVD* cardiovascular death, *HF* heart failure, *HR* hazard ratio, *MACE* major adverse cardiovascular events, *MI* myocardial infarction, *SGLT1/2* sodium–glucose cotransporter 1/2.

**Figure 2:**
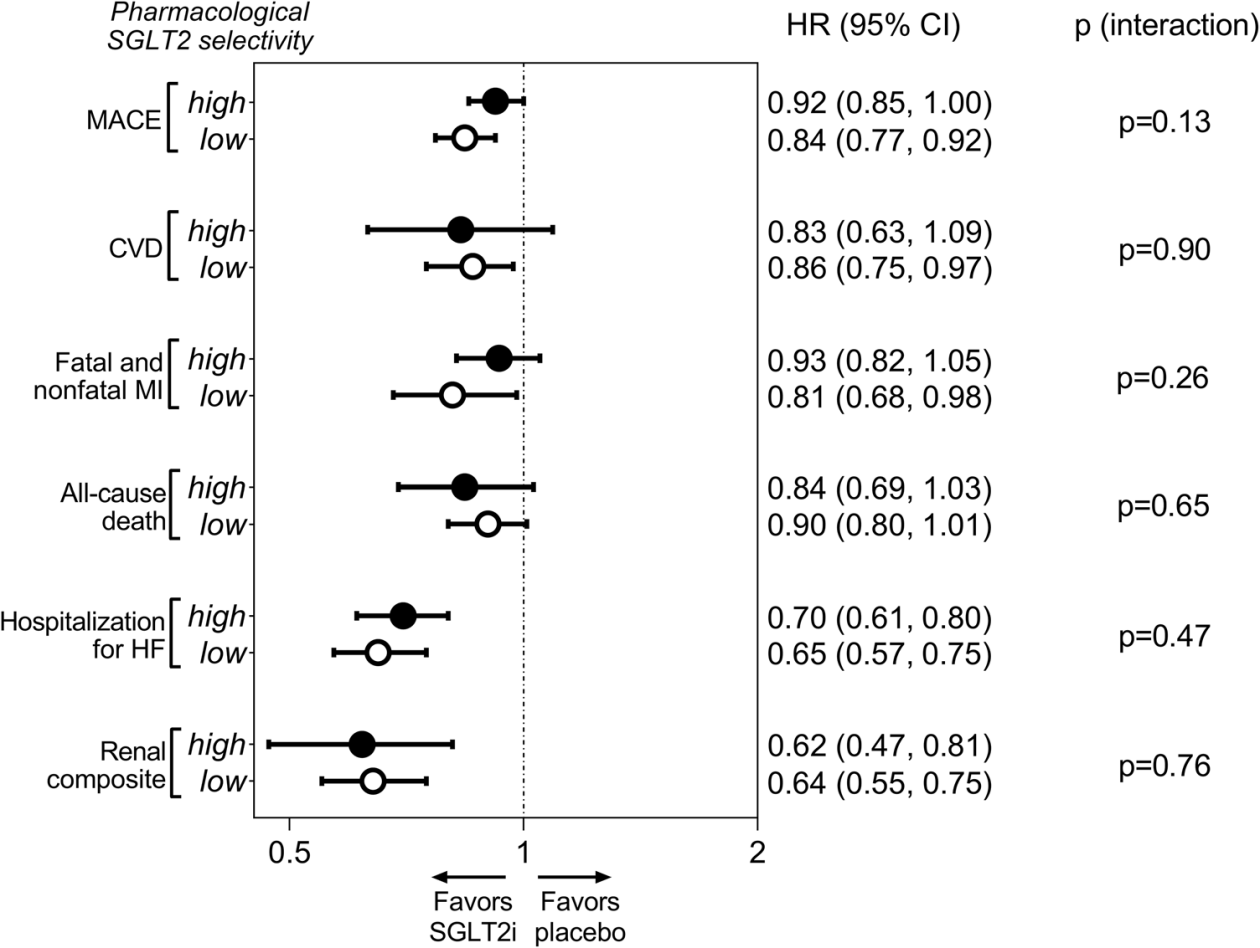
Comparison of the effect of high versus low pharmacological selectivity of SGLT2 inhibitors on clinical outcomes. *CI* confidence interval, *CVD* cardiovascular death, *HF* heart failure, *HR* hazard ratio, *MACE* major adverse cardiovascular events, *MI* myocardial infarction, *SGLT1/2* sodium–glucose cotransporter 1/2, *SGLT2i* sodium–glucose cotransporter 2 inhibitor.

Overall, SGLT2 inhibitors significantly reduced the relative risk of MACE compared with placebo (HR, 0.88; 95% CI, 0.83–0.95; p < 0.001) without relevant heterogeneity (I^2^ = 31%; p = 0.45) (Supplementary Fig. 2). The SGLT2:SGLT1 selectivity ratio of individual study medications as a continuous variable had no notable effect on MACE (pseudo-R^2^ = 13%; p = 0.31) (Fig. 1). When trials were grouped according to the pharmacological selectivity of the trial medications, low selectivity agents significantly reduced MACE versus placebo (HR, 0.84; 95% CI, 0.77–0.92), whereas in case of highly selective agents, the confidence interval crossed the line of unity (HR, 0.92; 95% CI, 0.85–1.00) (Fig. 2). However, there was no significant difference between these two pharmacological groups (p = 0.13) (Fig. 2).

Altogether, SGLT2 inhibitor treatment reduced the risk of cardiovascular death compared with placebo (HR, 0.84; 95% CI, 0.74–0.97; p = 0.014) with substantial heterogeneity (I^2^ = 62%; p = 0.043) (Supplementary Fig. 2), which was not explained by differences in selectivity of SGLT2 inhibitors to SGLT2 over SGLT1 (pseudo-R^2^ = 0%; p = 0.38) (Fig. 1). Accordingly, while only non-selective SGLT2 inhibitors reduced significantly the risk of cardiovascular death (HR, 0.86; 95% CI, 0.75–0.97) as compared with highly selective agents (HR, 0.83; 95% CI, 0.63–1.09) (Supplementary Fig. 2), there was no significant interaction (p = 0.90) (Fig. 2).

Fatal and nonfatal MI was slightly, but significantly reduced by SGLT2 inhibitors overall (HR, 0.88; 95% CI, 0.78–0.99; p = 0.031), with moderate heterogeneity (I^2^ = 47%; p = 0.26) (Supplementary Fig. 2). The ratio of pharmacological selectivity of SGLT2 inhibitors to SGLT2 over SGLT1 did not significantly correlate with clinical outcomes (pseudo-R^2^ = 18%; p = 0.24) (Fig. 1). Only non-selective SGLT2 inhibitors reduced significantly the risk of fatal and nonfatal MI compared with placebo (HR, 0.81; 95% CI, 0.68–0.98), whereas highly selective agents did not (HR, 0.93; 95% CI, 0.82–1.05), but there was no significant difference between the two groups (p = 0.26) (Fig. 2).

Across all 6 trials, the risk of all-cause death was significantly reduced by SGLT2 inhibitors (HR, 0.87; 95% CI, 0.78–0.97; p = 0.013), while heterogeneity was significant (I^2^ = 63%; p = 0.049) (Supplementary Fig. 3). Pharmacological selectivity of SGLT2 inhibitors did not affect all-cause death (pseudo-R^2^ = 10%; p = 0.25) (Fig. 1). Indeed, neither non-selective (HR, 0.90; 95% CI, 0.80–1.01) nor highly selective (HR, 0.84; 95% CI, 0.69–1.03) SGLT2 inhibitors reduced all-cause death as compared with placebo (p = 0.65) (Fig. 2).

In all trials, SGLT2 inhibitors significantly reduced the risk of hospitalization for HF with large effect size (HR, 0.68; 95% CI, 0.62–0.75; p < 0.001) and no heterogeneity (I^2^ = 5%; p = 0.92) (Supplementary Fig. 3). The SGLT2:SGLT1 pharmacological selectivity ratio did not correlate with this outcome (pseudo-R^2^ = 0%; p = 0.78) (Fig. 1). Agents with high (HR, 0.70; 95% CI, 0.61–0.80) and low (HR, 0.65; 95% CI, 0.57–0.75) SGLT2 selectivity reduced risk of HF hospitalization to a similar extent, with no significant difference (p = 0.47) (Fig. 2).

Next, SGLT2 inhibitors significantly reduced the risk of the composite renal endpoint (HR, 0.63; 95% CI, 0.54–0.73; p < 0.001), referring to a large effect with moderate heterogeneity (I^2^ = 46%; p = 0.16) (Supplementary Fig. 3). The extent of SGLT2 selectivity did not significantly affect this outcome (pseudo-R^2^ = 0%; p = 0.98) (Fig. 1). In fact, both highly selective (HR, 0.62; 95% CI, 0.47–0.81) and non-selective (HR, 0.64; 95% CI, 0.55–0.75) SGLT2 inhibitors significantly reduced the risk of the renal endpoint to a similar magnitude (p = 0.76) (Fig. 2).

**Figure 3:**
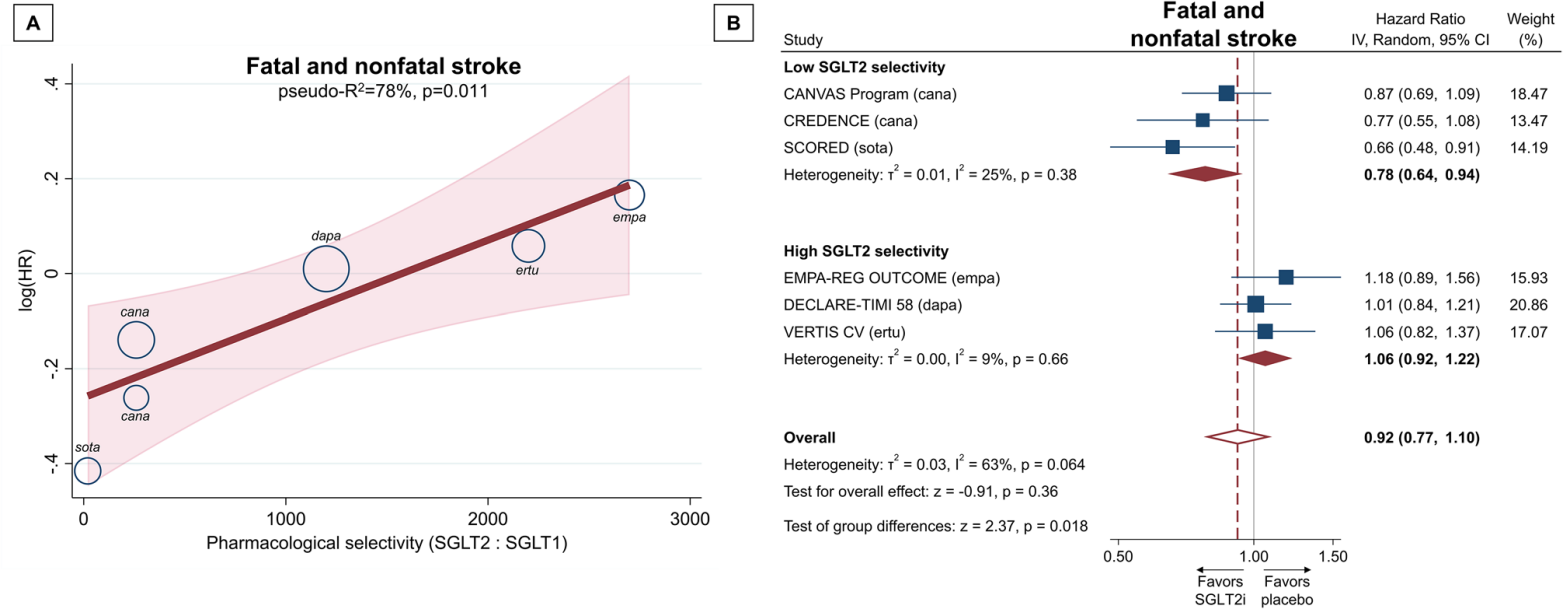
Effect of pharmacological selectivity of SGLT2 inhibitors on risk of fatal and nonfatal stroke either as continuous (SGLT2:SGLT1 pharmacological selectivity ratio) (**A**) or as binary (high vs. low SGLT2 selectivity) (**B**) explanatory variable. *CI* confidence interval, *HR* hazard ratio, *IV* inverse variance, *SGLT1/2* sodium–glucose cotransporter 1/2, *SGLT2i* sodium–glucose cotransporter 2 inhibitor.

Altogether, SGLT2 inhibitors did not alter the risk of fatal and nonfatal stroke in high-risk type 2 diabetic patients (HR, 0.92; 95% CI, 0.77–1.10; p = 0.36), but there was a substantial heterogeneity (I^2^ = 63%; p = 0.064) (Fig. 3B). The SGLT2:SGLT1 pharmacological selectivity ratio explained a considerable amount of heterogeneity in the risk of stroke (pseudo-R^2^ = 78%; p = 0.011) (Fig. 3A). Accordingly, less selectivity towards SGLT2 (i.e. more pronounced SGLT1 inhibitory effect) favored lower risk of stroke (Fig. 3A). In fact, only non-selective SGLT2 inhibitors reduced the risk of fatal and nonfatal stroke (HR, 0.78; 95% CI, 0.64–0.94) as compared with placebo, whereas those with high selectivity did not (HR, 1.06; 95% CI, 0.92–1.22) (Fig. 3B), with a significant interaction between the two pharmacological groups (p = 0.018). In a head-to-head comparison, this refers to a ~ 26% relative risk reduction in stroke with non-selective versus selective SGLT2 inhibitors (HR, 0.74; 95% CI, 0.58–0.93).

#### Safety

**Figure 4:**
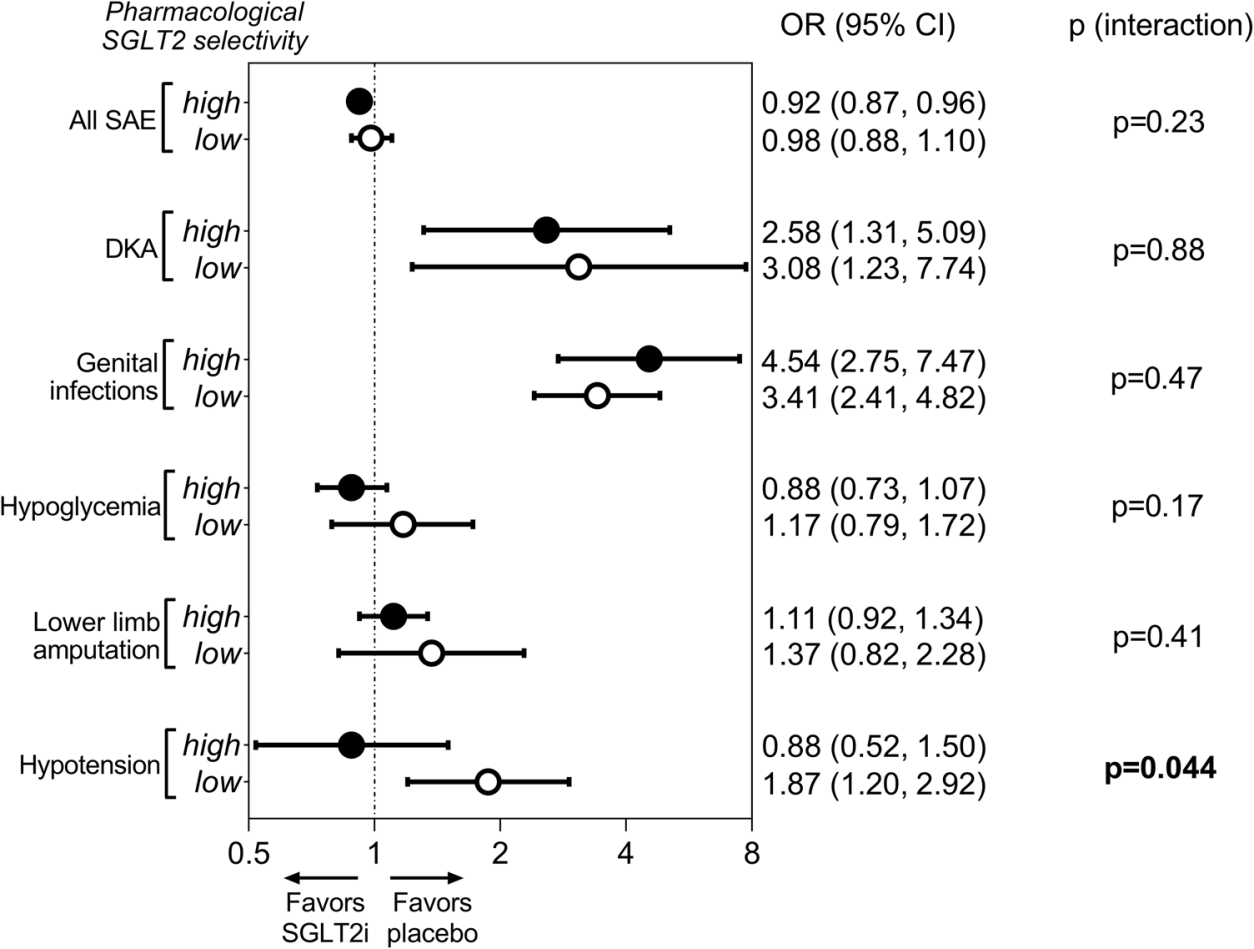
Comparison of the effect of high versus low pharmacological selectivity of SGLT2 inhibitors on safety outcomes. *CI* confidence interval, *DKA* diabetic ketoacidosis, *HR* hazard ratio, *SAE* severe adverse events, *SGLT2* sodium–glucose cotransporter 2, *SGLT2i* sodium–glucose cotransporter 2 inhibitor.

Both highly selective and non-selective SGLT2 inhibitors appeared to be safe compared with placebo. In fact, agents with high SGLT2 selectivity reduced the risk of severe adverse events (OR, 0.92; 95% CI, 0.87–0.96) whereas non-selective agents did not (OR, 0.98; 95% CI, 0.88–1.10), but there was no significant between-group difference (p = 0.23) (Fig. 4 and Supplementary Fig. 4). As compared with placebo, non-selective and highly selective SGLT2 inhibitors were associated with significantly higher risk of diabetic ketoacidosis (OR, 3.08; 95% CI, 1.23–7.74 vs. OR, 2.58; 95% CI, 1.31–5.09; p = 0.88) and genital infections (OR, 3.41; 95% CI, 2.41–4.82 vs. OR, 4.54; 95% CI, 2.75–7.47; p = 0.47), respectively, to a similar extent (Fig. 4 and Supplementary Figs. 5, 6). On the contrary, the risk of hypoglycemia (OR, 1.17; 95% CI, 0.79–1.72 vs. OR, 0.88; 95% CI, 0.73–1.07; p = 0.17) and lower limb amputation (OR, 1.37; 95% CI, 0.82–2.28 vs. OR, 1.11; 95% CI, 0.92–1.34; p = 0.41), respectively, was not significantly altered by these agents compared with placebo (Fig. 4 and Supplementary Figs. 7, 8). However, non-selective SGLT2 inhibitors significantly increased the risk of hypotension compared with placebo (HR, 1.87; 95% CI, 1.20–2.92), whereas highly selective agents did not (HR, 0.88; 95% CI, 0.52–1.50), with a significant between-group difference (p = 0.044) (Fig. 4 and Supplementary Fig. 9). In fact, less selectivity towards SGLT2 (i.e. more pronounced SGLT1 inhibitory effect) was associated with higher risk of hypotension (pseudo-R^2^ = 65%; p = 0.015) (Supplementary Fig. 9). The risk of hypotension was ~ 2-times higher with non-selective inhibitors as compared with highly selective SGLT2 inhibitors (OR, 2.13; 95% CI, 1.06–4.24).

### Sensitivity analysis

For fatal and nonfatal stroke, we analyzed data of patients with baseline eGFR lower than 60 mL/min/1.73 m^2^ pooling data from a previous meta-analysis^37^. Data from the VERTIS CV trial^6^ were unavailable for this analysis. In patients with eGFR lower than 60 mL/min/1.73 m^2^, SGLT2 inhibitors tended to reduce the risk of stroke (HR, 0.75; 95% CI, 0.55–1.02; p = 0.066) with some heterogeneity (I^2^ = 58%; p = 0.14) (Supplementary Fig. 10). Compared to agents with high SGLT2 selectivity, low SGLT2 selectivity was associated with a significantly lower risk of stroke (HR, 0.63; 95% CI, 0.48–0.81) in patients with eGFR lower than 60 mL/min/1.73 m^2^, marking a significant difference between the two pharmacological groups (p = 0.047) (Supplementary Fig. 10).

## Discussion

In this meta-analysis, we investigated the impact of pharmacological selectivity of SGLT2 inhibitors on cardiovascular outcomes in patients with type 2 diabetes mellitus, according to large-scale cardiovascular outcome trials. We found that a more pronounced SGLT1 inhibitory property had little additional effect on most outcomes, however, it was significantly associated with lower risk of stroke as compared with high pharmacological SGLT2 selectivity. In terms of adverse events, the risk of hypotension appears to be higher with non-selective SGLT2 inhibitors.

The substantial differences between the extent of pharmacological selectivity of SGLT2 inhibitors to SGLT2 over SGLT1 might be clinically relevant since individuals with partially reduced transport activity of SGLT1 (corresponding to pharmacological SGLT1 inhibition), but not that of SGLT2, derive significant cardiovascular and survival benefits as compared with non-affected controls^23,24^. A recent meta-analysis of dedicated HF studies found that lower SGLT2 selectivity was significantly more favorable in terms of the composite of hospitalization for HF or cardiovascular death^38^. In line with these clinical data, a number of preclinical studies have linked SGLT1 to pathological processes in the heart^25–29^, brain^30,31^, and kidney^32^. Yet, in high-risk type 2 diabetic patients, the effect of the pharmacological selectivity of SGLT2 inhibitors on clinical outcomes has been ill-defined.

Here we report that pharmacological selectivity of SGLT2 inhibitors does not significantly correlate with risk of MACE, cardiovascular death, fatal and nonfatal MI, all-cause mortality, hospitalization for HF, or the renal composite outcome in patients with type 2 diabetes mellitus and high cardiovascular risk. Therefore, it seems that additional SGLT1 inhibition on top of SGLT2 blockade might not affect these outcomes in the studied patient groups. On the contrary, pharmacological SGLT2:SGLT1 selectivity ratio significantly correlated with stroke outcomes, with lower SGLT2 selectivity (i.e. more pronounced inhibitory effect on SGLT1) corresponding with reduced risk. In addition, this difference remained significant even in patients with a baseline eGFR lower than 60 mL/min/1.73 m^2^. Therefore, SGLT2 inhibitors with pronounced SGLT1 inhibitory effect might reduce the risk of stroke, which effect seems to be less affected by baseline eGFR.

Previous meta-analyses of large cardiovascular outcome trials^39,40^ found that SGLT2 inhibitors have only modest effect on atherosclerotic major adverse cardiovascular outcomes in patients with type 2 diabetes mellitus, and this is confined to those with established atherosclerotic cardiovascular disease (ASCVD). Specifically, the risk of MACE and (fatal and nonfatal) MI, respectively, were shown to be significantly reduced by SGLT2 inhibitors only in type 2 diabetic patients with established ASCVD, but not in those without ASCVD^39,40^. However, SGLT2 inhibitors had neutral effect on stroke risk in patients with and without ASCVD^39^. Therefore, our present results might complement previous meta-analyses by adding that selectivity of SGLT2 inhibitors is a significant predictor of stroke outcomes, in fact, combined SGLT1/2 inhibition might constitute a novel pharmacological approach to reduce adverse stroke outcomes in type 2 diabetic patients who are inherently at greater risk.

Ischemic stroke was the predominant subtype in these outcome trials, and SGLT2 inhibitors overall reduce new onset atrial fibrillation or flutter (AF/AFL)^37^, a major risk factor for ischemic stroke. On individual trial level, only dapagliflozin (DECLARE-TIMI 58 trial) reduced the risk of AF/AFL, which was independent of patient’s previous history of AF, ASCVD, or HF^4,41^. However, dapagliflozin had neutral effect on risk of ischemic stroke^4^. Therefore, it is unlikely that SGLT2 inhibitor treatment alters stroke risk by reducing new onset AF/AFL occurrence. Other SGLT1-related mechanisms have recently been suggested by Pitt and colleagues^20^, including favorable alteration of gut microbiome due to intestinal inhibition of SGLT1-mediated glucose absorption, and increased native glucagon-like peptide-1 levels, both having direct and indirect antithrombotic effects, and at the same time, postprandial serum glucose excursions are blunted^20^. Interestingly, small animal studies have linked SGLT1 upregulation during ischemic stroke to neuronal damage possibly through enhanced glucose uptake, its knockdown reduced lesion volume^30^ and brain injury^31^. Currently, it is unclear whether pharmacological inhibition of cerebral SGLT1 itself has any clinical relevance and whether it plays any role in prevention of stroke.

Regarding safety outcomes, both highly selective and non-selective SGLT2 inhibitors increased the risk of genital infections and diabetic ketoacidosis to a similar extent in patients with type 2 diabetes, as compared with placebo. On the contrary, neither pharmacological subgroup heightened significantly the risk of hypoglycemia or lower limb amputation. However, non-selective SGLT2 inhibitors were associated with a twofold increased risk of hypotension compared with highly selective agents. This might again reflect on distinct mechanistic effects of selective versus non-selective SGLT2 inhibitors.

In summary, we found that lower pharmacological selectivity of SGLT2 inhibitors with a more pronounced inhibitory effect on SGLT1 is associated with reduced risk of fatal and nonfatal stroke in high-risk type 2 diabetic patients, according to large-scale cardiovascular outcome trials. Combined SGLT1/2 inhibition could be a novel pharmacological approach to prevent stroke in these patients. Future confirmatory studies are warranted to elucidate the clinical significance of these hypothesis-generating findings.

### Limitations

Our meta-analysis has inherent limitations, rendering the findings hypothesis-generating only. The included trials were not powered to assess individual endpoints of the composite outcome. The number of studies included is relatively small, however, these trials enrolled a relatively high number of patients. Differences in eligibility criteria and baseline characteristics may have affected the calculations, but the included cardiovascular outcome trials similarly enrolled type 2 diabetic patients with high cardiovascular risk. Definitions of outcomes were slightly different across trials. The definition of the renal composite varied significantly, limiting the interpretation of this endpoint. The DECLARE-TIMI 58^4^ trial reported fatal and nonfatal stroke of ischemic origin only, however the incidence of hemorrhagic stroke was only 0.09% in the overall trial population, suggesting that the composite endpoint would not meaningfully change if hemorrhagic stroke was included. The EMPA-REG OUTCOME^2^ trial excluded silent MI from the endpoints of ‘MACE’ and ‘fatal and nonfatal MI’, respectively. Finally, the SCORED trial^7^ was terminated prematurely due to loss of funding, therefore the primary endpoint was changed during the trial and investigator-reported events were used for endpoint analyses.

### Supplementary Information

Supplementary Information.

## Data Availability

Original data generated and analyzed during this study are included in this manuscript.
