# Supplementary material for: Effect of pharmacological selectivity of SGLT2 inhibitors on cardiovascular outcomes in patients with type 2 diabetes: a meta-analysis
Source: Sci Rep. 2024 Jan 25;14:2188. doi: 10.1038/s41598-024-52331-w (PMC10810805; doi:10.1038/s41598-024-52331-w)
Supplement: Supplementary file 1 — Supplementary Information. [file 41598_2024_52331_MOESM1_ESM.docx]

**Supplementary materials**

# 1. Search terms

**PubMed search terms as of 1^st^ of September 2022:**

((("SGLT"[All Fields] AND ("antagonists and inhibitors"[MeSH Subheading] OR ("antagonists"[All Fields] AND "inhibitors"[All Fields]) OR "antagonists and inhibitors"[All Fields] OR "inhibitors"[All Fields] OR "inhibitor"[All Fields] OR "inhibitor s"[All Fields])) OR ("empagliflozin"[Supplementary Concept] OR "empagliflozin"[All Fields]) OR ("canagliflozin"[MeSH Terms] OR "canagliflozin"[All Fields]) OR ("dapagliflozin"[Supplementary Concept] OR "dapagliflozin"[All Fields] OR "dapagliflozin s"[All Fields]) OR ("ertugliflozin"[Supplementary Concept] OR "ertugliflozin"[All Fields]) OR ("2s 3r 4r 5s 6r 2 4 chloro 3 4 ethoxybenzyl phenyl 6 methylthio tetrahydro 2h pyran 3 4 5 triol"[Supplementary Concept] OR "2s 3r 4r 5s 6r 2 4 chloro 3 4 ethoxybenzyl phenyl 6 methylthio tetrahydro 2h pyran 3 4 5 triol"[All Fields] OR "sotagliflozin"[All Fields])) AND (("cardiovascular system"[MeSH Terms] OR ("cardiovascular"[All Fields] AND "system"[All Fields]) OR "cardiovascular system"[All Fields] OR "cardiovascular"[All Fields] OR "cardiovasculars"[All Fields]) AND ("event"[All Fields] OR "event s"[All Fields] OR "events"[All Fields])) AND ("diabete"[All Fields] OR "diabetes mellitus"[MeSH Terms] OR ("diabetes"[All Fields] AND "mellitus"[All Fields]) OR "diabetes mellitus"[All Fields] OR "diabetes"[All Fields] OR "diabetes insipidus"[MeSH Terms] OR ("diabetes"[All Fields] AND "insipidus"[All Fields]) OR "diabetes insipidus"[All Fields] OR "diabetic"[All Fields] OR "diabetics"[All Fields] OR "diabets"[All Fields]) AND "randomized controlled trial"[Publication Type]) AND (randomizedcontrolledtrial[Filter])

**Clinicaltrials.gov search terms as of 1^st^ of September 2022:**

Condition: type 2 diabetes

Other terms: sodium glucose cotransporter OR SGLT OR gliflozin OR sodium-glucose transporter 2 inhibitors OR sodium glucose transporter

Study type: interventional studies (clinical trials)

Status (recruitment): completed

Age: adult (18-64), older adult (65+)

Outcome measure: cardiovascular

# 2. Supplementary table

**Supplementary Table 1.** Risk of bias assessment of included clinical trials.

|  | Randomization process | Deviations from intended interventions | Missing outcome data | Measurement of outcome | Selection of the reported results |
| --- | --- | --- | --- | --- | --- |
| EMPA-REG OUTCOME | low | low | low | low | low |
| CANVAS Program | low | low | low | low | low |
| DECLARE-TIMI 58 | low | low | low | low | low |
| CREDENCE | low | low | low | low | low |
| VERTIS CV | low | low | low | low | low |
| SCORED | low | low | low | ? | low |

# 3. Supplementary figure legends

**Supplementary Figure 1.** Selection process of eligible studies included in the meta-analyses based on prespecified inclusion and exclusion criteria.

MACE=major adverse cardiovascular events (composite of cardiovascular death, nonfatal myocardial infarction, and nonfatal stroke)

**Supplementary Figure 2.** Effect of pharmacological selectivity of sodium-glucose cotransporter 2 (SGLT2) inhibitors on major adverse cardiovascular events (MACE, the composite of cardiovascular death, nonfatal myocardial infarction [MI], and nonfatal stroke) (**A**), cardiovascular death (**B**), and fatal and nonfatal MI (**C**).

Trials are dichotomized based on whether the given SGLT2 inhibitor has clinically relevant SGLT1 inhibitory effect (‘low SGLT2 selectivity’: canagliflozin, sotagliflozin) or not (‘high SGLT2 selectivity’: empagliflozin, dapagliflozin, ertugliflozin). Mixed-effects meta-regression analysis was applied to explore interaction between these two groups.

CI=confidence interval; IV=inverse variance; SGLT1/2=sodium-glucose cotransporter 1/2

**Supplementary Figure 3.** Effect of pharmacological selectivity of sodium-glucose cotransporter 2 (SGLT2) inhibitors on all-cause death (**A**), hospitalization for heart failure (HF) (**B**), and renal composite endpoint (**C**).

Trials are dichotomized based on whether the given SGLT2 inhibitor has clinically relevant SGLT1 inhibitory effect (‘low SGLT2 selectivity’: canagliflozin, sotagliflozin) or not (‘high SGLT2 selectivity’: empagliflozin, dapagliflozin, ertugliflozin). Mixed-effects meta-regression analysis was applied to explore interaction between these two groups.

CI=confidence interval; IV=inverse variance; SGLT1/2=sodium-glucose cotransporter 1/2

**Supplementary Figure 4.** Effect of pharmacological selectivity of sodium-glucose cotransporter 2 (SGLT2) inhibitors on risk of severe adverse events.

Trials are dichotomized based on whether the given SGLT2 inhibitor has clinically relevant SGLT1 inhibitory effect (‘low SGLT2 selectivity’: canagliflozin, sotagliflozin) or not (‘high SGLT2 selectivity’: empagliflozin, dapagliflozin, ertugliflozin). Mixed-effects meta-regression analysis was applied to explore interaction between these two groups.

CI=confidence interval; IV=inverse variance; SAE=severe adverse events; SGLT2=sodium-glucose cotransporter 2

**Supplementary Figure 5.** Effect of pharmacological selectivity of sodium-glucose cotransporter 2 (SGLT2) inhibitors on risk of diabetic ketoacidosis.

Trials are dichotomized based on whether the given SGLT2 inhibitor has clinically relevant SGLT1 inhibitory effect (‘low SGLT2 selectivity’: canagliflozin, sotagliflozin) or not (‘high SGLT2 selectivity’: empagliflozin, dapagliflozin, ertugliflozin). Mixed-effects meta-regression analysis was applied to explore interaction between these two groups.

CI=confidence interval; DKA=diabetic ketoacidosis; IV=inverse variance; SGLT2=sodium-glucose cotransporter 2

**Supplementary Figure 6.** Effect of pharmacological selectivity of sodium-glucose cotransporter 2 (SGLT2) inhibitors on risk of genital infections.

Trials are dichotomized based on whether the given SGLT2 inhibitor has clinically relevant SGLT1 inhibitory effect (‘low SGLT2 selectivity’: canagliflozin, sotagliflozin) or not (‘high SGLT2 selectivity’: empagliflozin, dapagliflozin, ertugliflozin). Mixed-effects meta-regression analysis was applied to explore interaction between these two groups.

CI=confidence interval; IV=inverse variance; SGLT2=sodium-glucose cotransporter 2

**Supplementary Figure 7.** Effect of pharmacological selectivity of sodium-glucose cotransporter 2 (SGLT2) inhibitors on risk of hypoglycemia.

Trials are dichotomized based on whether the given SGLT2 inhibitor has clinically relevant SGLT1 inhibitory effect (‘low SGLT2 selectivity’: canagliflozin, sotagliflozin) or not (‘high SGLT2 selectivity’: empagliflozin, dapagliflozin, ertugliflozin). Mixed-effects meta-regression analysis was applied to explore interaction between these two groups.

CI=confidence interval; IV=inverse variance; SGLT2=sodium-glucose cotransporter 2

**Supplementary Figure 8.** Effect of pharmacological selectivity of sodium-glucose cotransporter 2 (SGLT2) inhibitors on risk of lower limb amputation.

Trials are dichotomized based on whether the given SGLT2 inhibitor has clinically relevant SGLT1 inhibitory effect (‘low SGLT2 selectivity’: canagliflozin, sotagliflozin) or not (‘high SGLT2 selectivity’: empagliflozin, dapagliflozin, ertugliflozin). Mixed-effects meta-regression analysis was applied to explore interaction between these two groups.

CI=confidence interval; IV=inverse variance; SGLT2=sodium-glucose cotransporter 2

**Supplementary Figure 9.** Effect of pharmacological selectivity of sodium-glucose cotransporter 2 (SGLT2) inhibitors as continuous (A) or dichotomized (B) variable on risk of hypotension.

Trials are dichotomized based on whether the given SGLT2 inhibitor has clinically relevant SGLT1 inhibitory effect (‘low SGLT2 selectivity’: canagliflozin, sotagliflozin) or not (‘high SGLT2 selectivity’: empagliflozin, dapagliflozin, ertugliflozin). Mixed-effects meta-regression analysis was applied to explore interaction between these two groups.

CI=confidence interval; IV=inverse variance; OR=odds ratio; SGLT1/2=sodium-glucose cotransporter 1/2

**Supplementary Figure 10.** Effect of SGLT2 inhibitors on fatal and nonfatal stroke in patients with estimated glomerular filtration rate (eGFR) lower than 60 mL/min/1.73m^2^, when trials are dichotomized based on whether the given SGLT2 inhibitor has clinically relevant SGLT1 inhibitory effect (‘low SGLT2 selectivity’: canagliflozin, sotagliflozin) or not (‘high SGLT2 selectivity’: empagliflozin, dapagliflozin). Mixed-effects meta-regression analysis was applied to explore interaction between these two groups. Data from the VERTIS CV trial was not available for this analysis.

CI=confidence interval, IV=inverse variance; SGLT1/2=sodium-glucose cotransporter 1/2

Supplementary Figure 1.


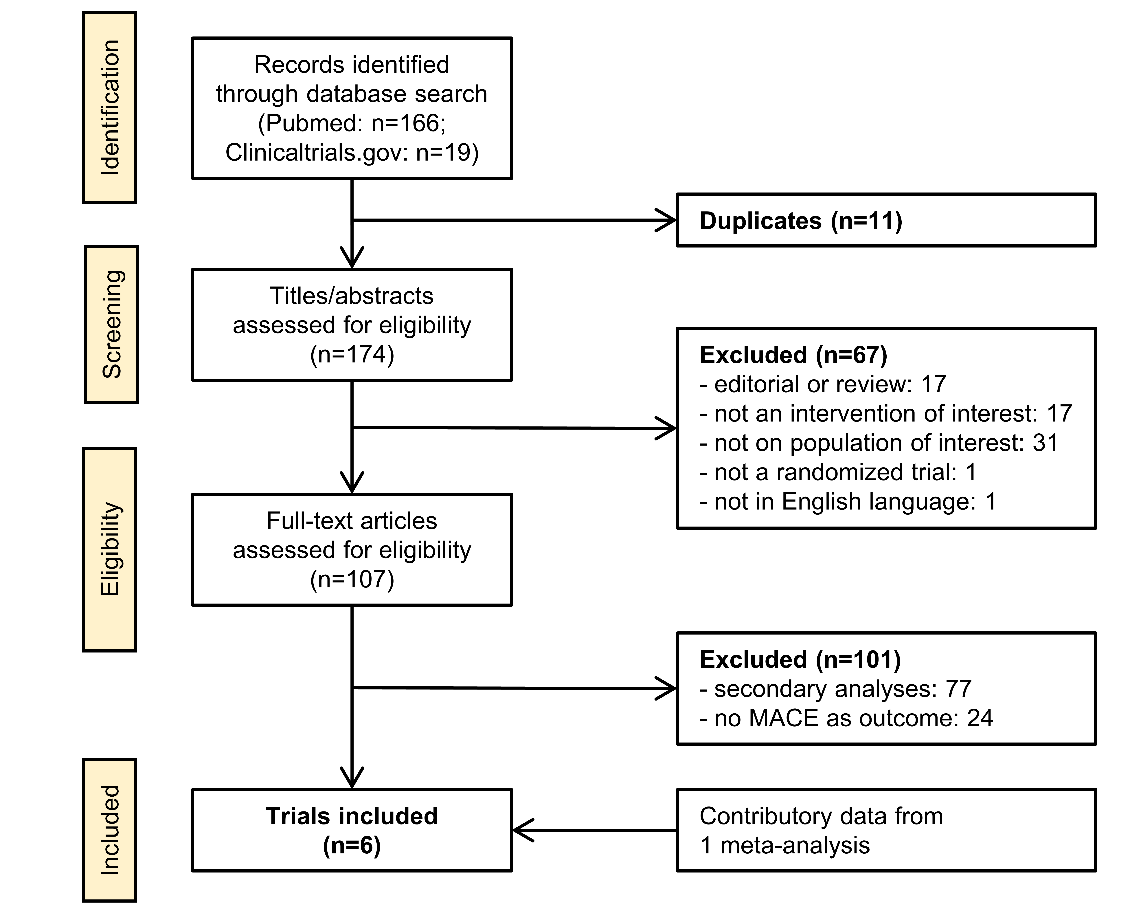



Supplementary Figure 2.



Supplementary Figure 3.

Supplementary Figure 4.


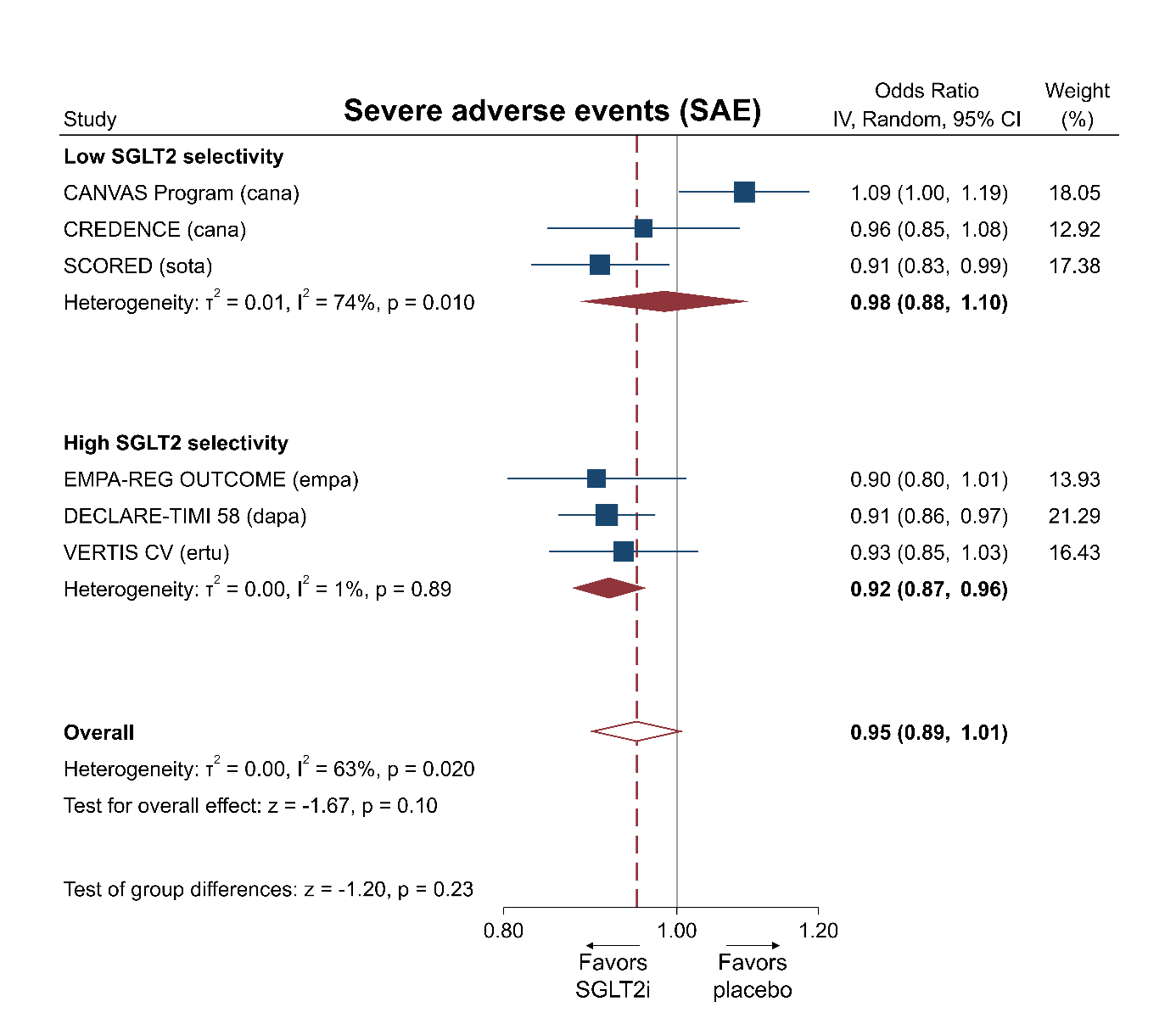


Supplementary Figure 5.


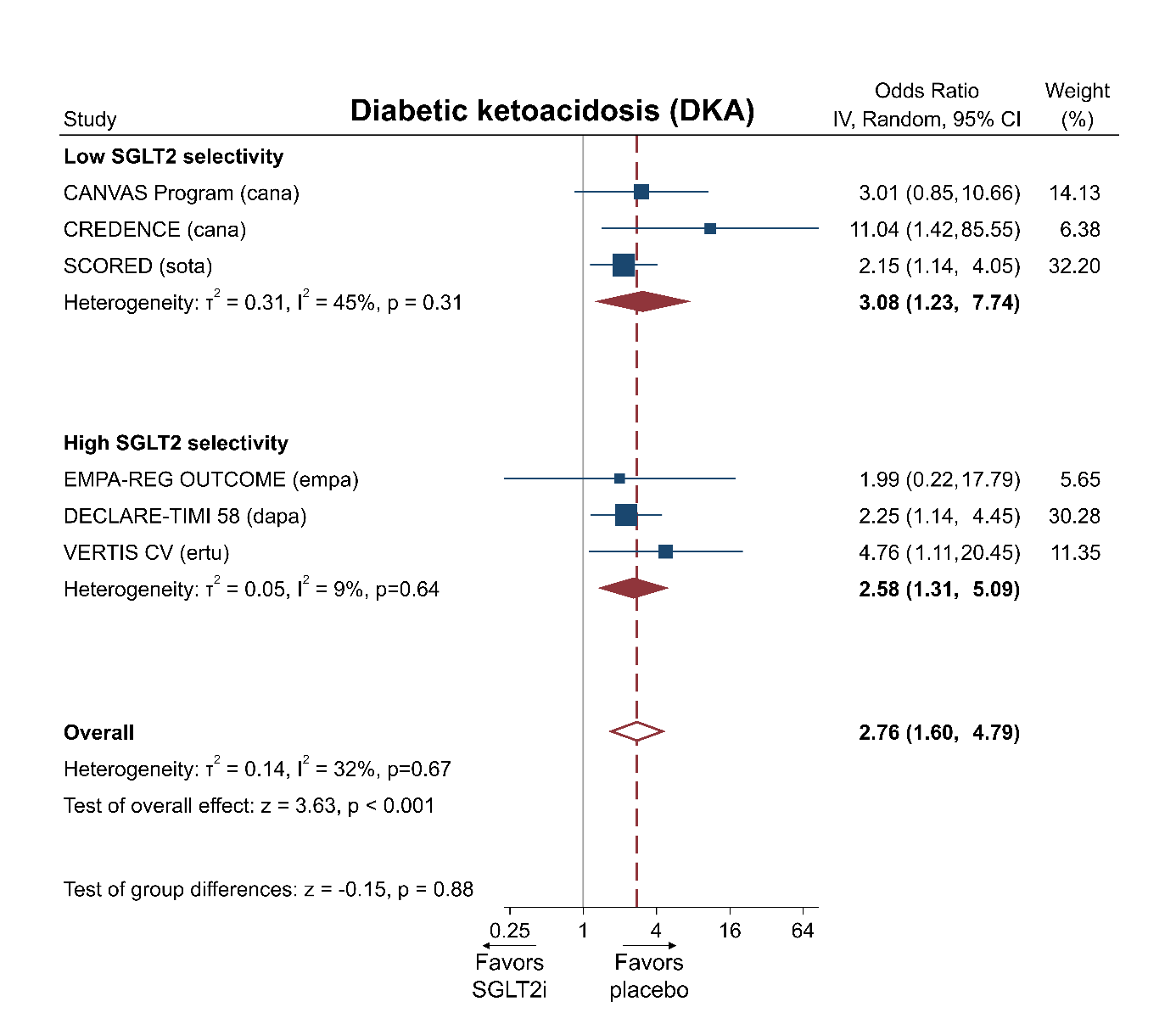


Supplementary Figure 6.


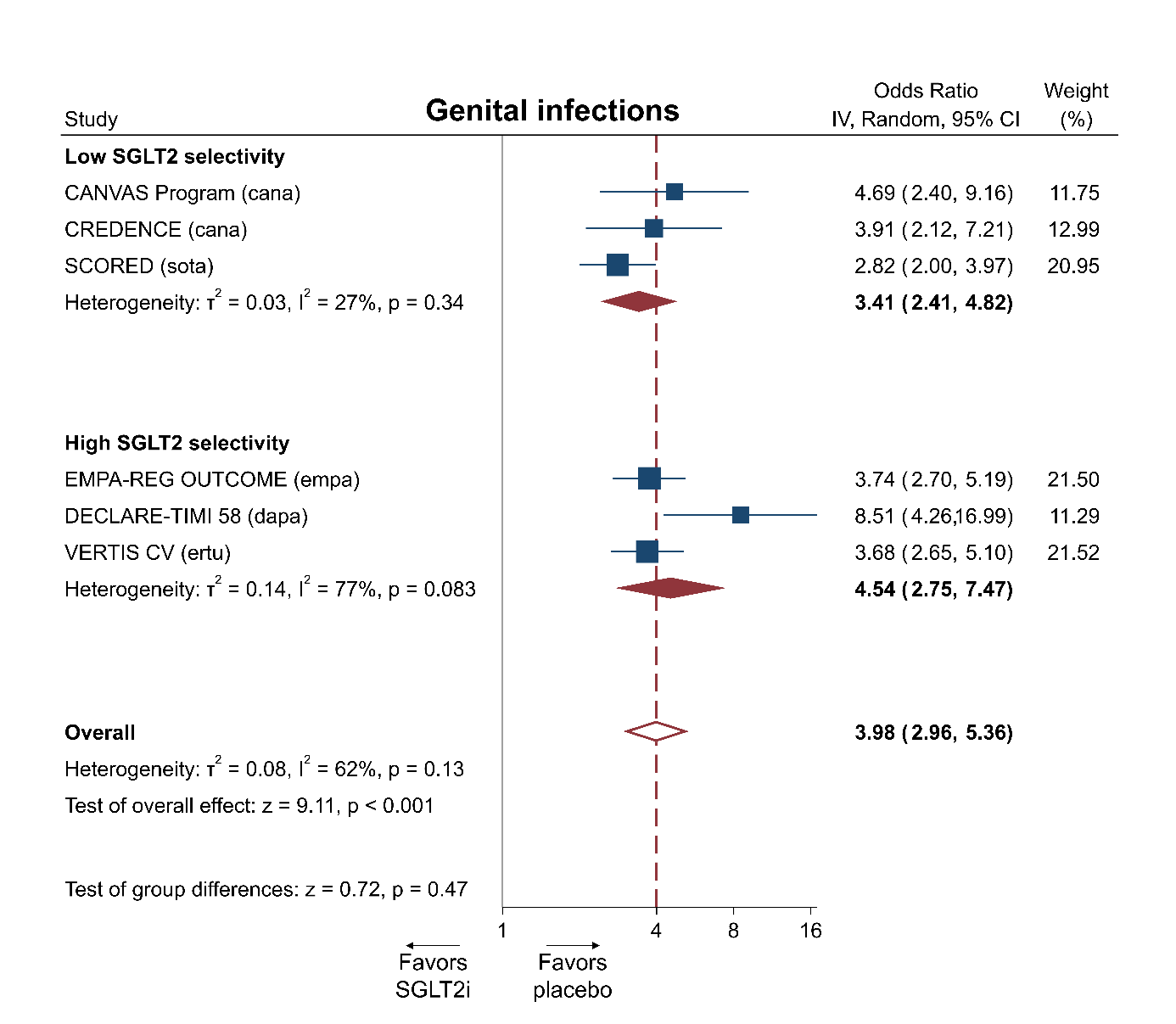


Supplementary Figure 7.


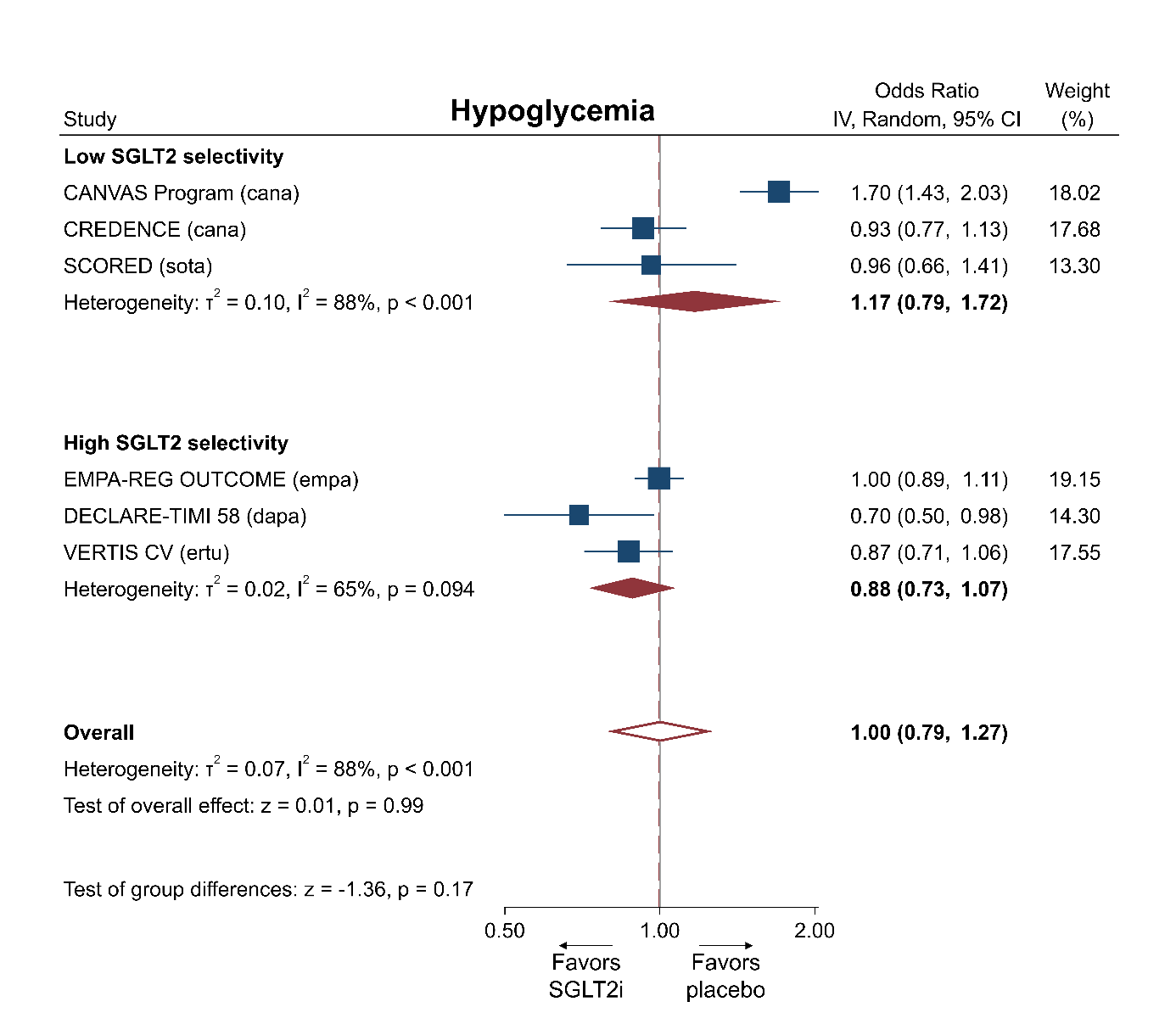


Supplementary Figure 8.


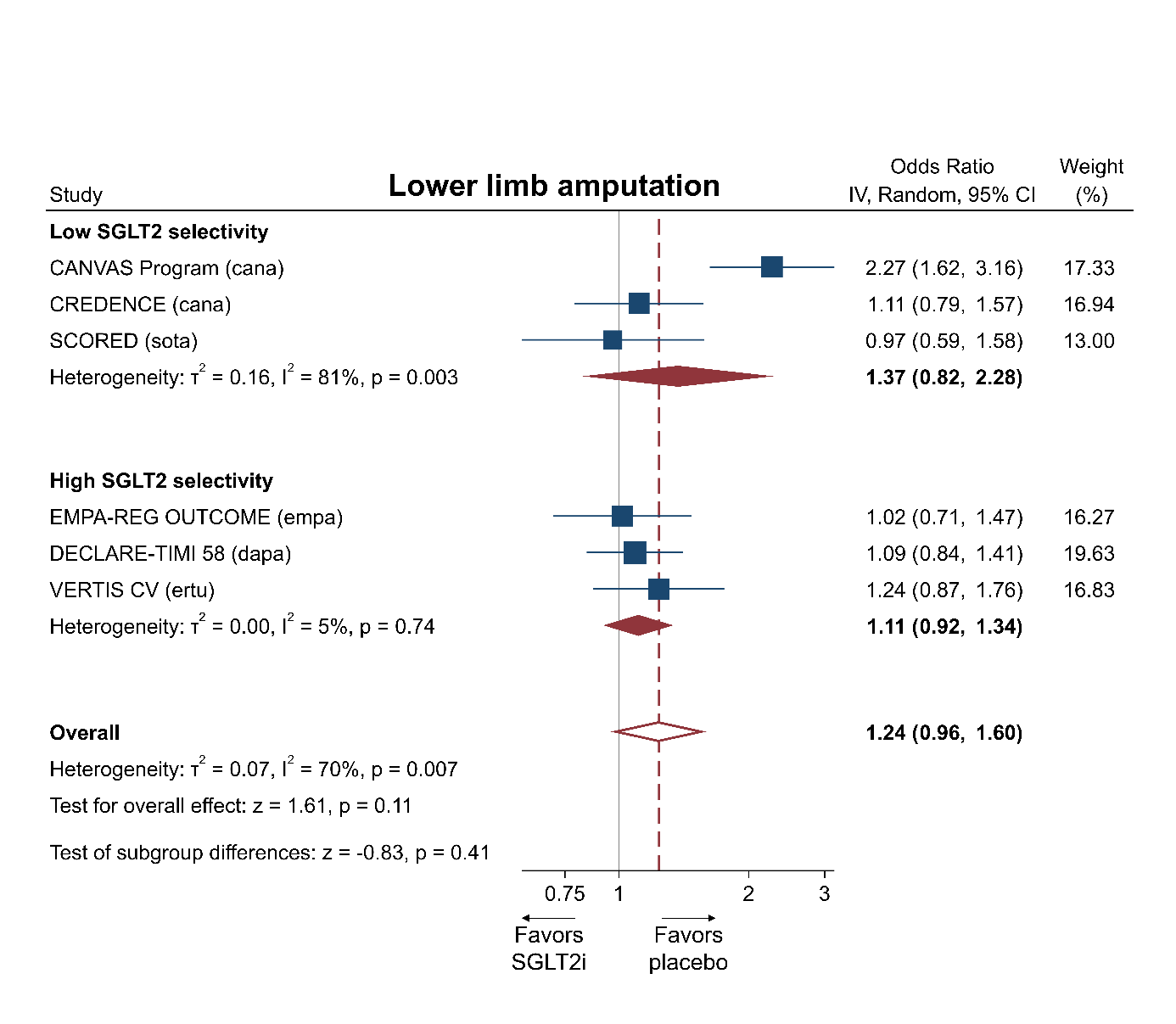


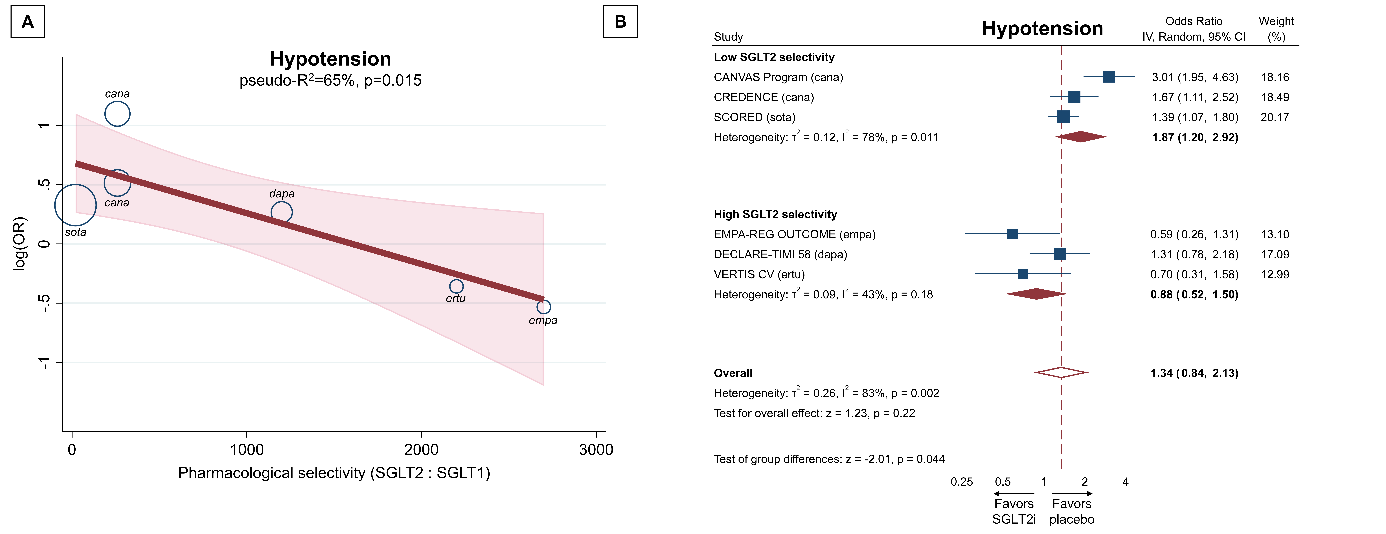
Supplementary Figure 9.


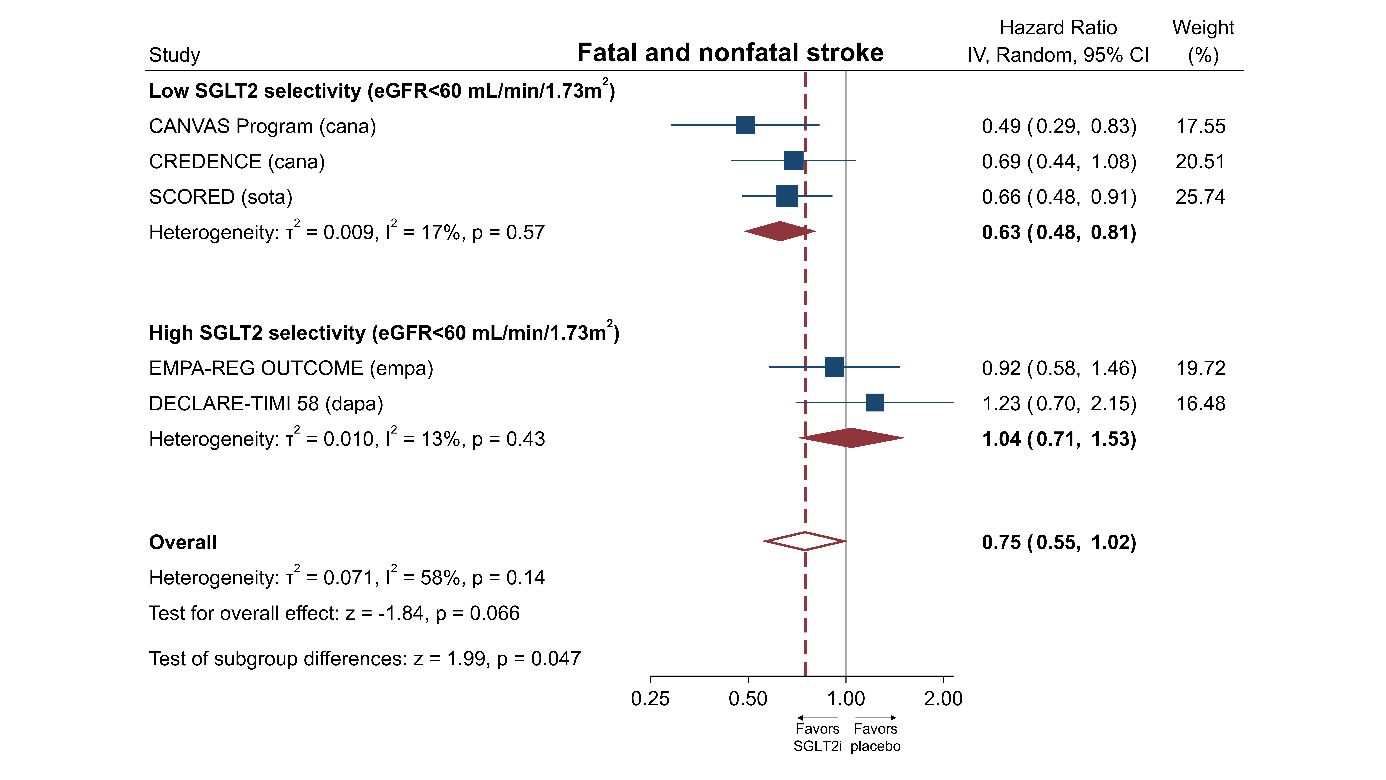
Supplementary Figure 10.
